# Determination of Ten Corticosteroids in Illegal Cosmetic Products by a Simple, Rapid, and High-Performance LC-MS/MS Method

**DOI:** 10.1155/2017/3531649

**Published:** 2017-02-14

**Authors:** Vita Giaccone, Giuseppe Polizzotto, Andrea Macaluso, Gaetano Cammilleri, Vincenzo Ferrantelli

**Affiliations:** Istituto Zooprofilattico Sperimentale della Sicilia “A. Mirri”, Via Gino Marinuzzi 3, 90129 Palermo, Italy

## Abstract

The aim of our present work was the development of a rapid high-performance liquid chromatography method with electrospray ionization and tandem mass spectrometry detection (LC-ESI-MS/MS) for the determination of several corticosteroids in cosmetic products. Corticosteroids are suspected to be illegally added in cosmetic preparations in order to enhance the curative effect against some skin diseases. Sample preparation step consists in a single extraction with acetonitrile followed by centrifugation and filtration. The compounds were separated by reversed-phase chromatography with water and acetonitrile (both with 0.1% formic acid) gradient elution and detected by ESI-MS positive and negative ionization mode. The method was validated at the validation level of 0.1 mg kg^−1^. Linearity was studied in the 5–250 *μ*g L^−1^ range and linear coefficients (*r*^2^) were all over 0.99. The accuracy and precision of the method were satisfactory. The LOD ranged from 0.085 to 0.109 mg kg^−1^ and the LOQ from 0.102 to 0.121 mg kg^−1^. Mean recoveries for all the analytes were within the range 91.9–99.2%. The developed method is sensitive and useful for detection, quantification, and confirmation of these corticosteroids in cosmetic preparations and can be applied in the analysis of the suspected samples under investigation.

## 1. Introduction

Corticosteroids are known to be highly effective drugs widely used for the treatment of inflammatory diseases. In dermatology, they were used for the treatment of skin disorders such as psoriasis, dermatoses, and eczema. They reduce inflammation and can temporarily relieve the symptoms of inflammatory skin problems of severe plaque psoriasis. For topical use they are available in the form of creams, gels, and ointments with different potency and efficacy.

Side effects and efficacy have to be related to their anti-inflammatory propriety, though no active principle shows better risks/benefits ratio compared to the others [1]. The best method to evaluate the potency of corticosteroids for topical usage is the vasoconstriction test, which allows assessing the vasoconstrictor effect induced by formulations for topical usage in healthy subjects [2]. However, such test is not quite accurate because it does not consider the treatment period and frequency nor the individual response [3]. Currently topical corticosteroids are classified into seven groups according to their potency [4]. Active principles with low potency can be used for a long time and on wide skin surface; conversely, the principles with high potency should be used for short time and not applied on sensitive skin areas, such as face and armpits [5].

Prolonged therapy with corticosteroids preparations may result in adverse effects like skin atrophy, cutaneous reactivity and some systematic side effects, hypertension, diabetes mellitus, osteoporosis, allergic contact dermatitis, Cushing's syndrome, and so forth [6].

For this reason, cosmetic products should not contain glucocorticoids; nevertheless, some cosmetic preparations intended for treatment of seborrhea or psoriasis are indicated as capable of giving improvement, without clearly showing the content of glucocorticoids. Cosmetic products have no therapeutic purposes and must not claim any therapeutic action [7]. For these reasons, consumers may have risk of experiencing side effects in case of a long-term use and high doses, especially without medical supervision [8, 9].

**Figure 1:**
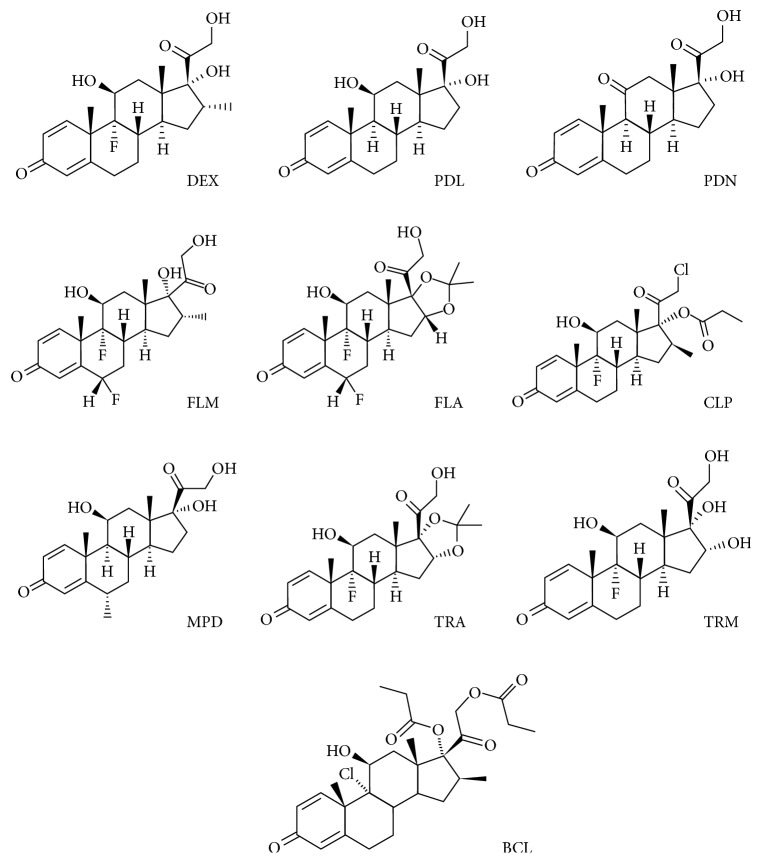
Chemical structures of corticosteroids.

Therefore, there is a need for an analytical method for rapid screening of cosmetic products such as creams, ointments, and gels, which are banned in the presence of glucocorticoids and sold without health care. Earlier papers reported a number of different LC methods for these steroids in biological matrices or pharmaceutical formulations [10–15]; the goal of this paper is to report the simultaneous determination of a pool of 10 different active ingredients using a simple and rapid method. In this study a simple UHPLC separation method with ESI-MS-MS detection for investigating the illegal presence of methylprednisolone (MPD), dexamethasone (DEX), prednisolone (PDL), fluocinolone acetonide (FLA), flumetasone (FLM), prednisone (PDN), triamcinolone (TRM), triamcinolone acetonide (TRA), beclomethasone (BCL), and clobetasol propionate (CLP) in cosmetic preparations was developed. We choose to investigate these specific corticosteroids because they are the most used ones in dermatologic field and to ensure that the investigation is carried out as widely as possible. In the above-mentioned classification, CLP 0.05% (v/v) is classified in class I, with a potency that is 1800 times higher compared to hydrocortisone [16]. DEX, FLM, MPD, and PDL are classified in class VII, with TRA classified in class III. FLA is classified in class VI. Chemical structures of the target analytes are reported in Figure 1.

Although it is known that these drugs, in the case of cosmetic products, should be present at comparable concentration of the pharmaceutical formulation to induce a pharmacologic effect, we choose a very sensitive and specific method such as LC-MS/MS to identify these analytes at very low concentrations. The method was validated for linearity, accuracy, precision, and sensitivity by analyzing different pharmaceutical formulations as complex matrices.

## 2. Materials and Methods

### 2.1. Chemicals, Solvents, and Samples

Methanol and acetonitrile and formic acid 99.9% (LC-MS grade) and water (HPLC gradient grade) were supplied from VWR (VWR International PBI Srl, Milan, Italy). Methylprednisolone, dexamethasone, prednisolone, fluocinolone acetonide, flumetasone, prednisone, triamcinolone, triamcinolone acetonide, beclomethasone, clobetasol propionate, dexamethasone D4, methylprednisolone D2, and prednisolone D6 (purity > 98%) were purchased from Sigma-Aldrich (Milan, Italy). A 1000 mg L^−1^ stock solution was made by dissolving the standard in methanol. From this solution, a 10 mg L^−1^ work solution was made by dilution in methanol.

### 2.2. Sample Preparation

About 2 g of the sample was mixed to attain a homogeneous mixture; 1 g of the homogenized mixture was accurately weighed into a 15 mL centrifuge tube. The sample was spiked with 100 *μ*L of the mixed solution of internal standard (IS) at 10 mg L^−1^ and then was treated with 10 mL of acetonitrile, shaken by vortex for 30 s and by automatic shaker for 10 minutes. The solution was centrifuged for 5 min at 4000 rpm and the supernatant was filtered through a 0.45 mm cellulose acetate filter. Finally, the solution containing the sample was transferred into a 1 mL vial.

### 2.3. Chromatographic Conditions

LC analysis was carried out through a Thermo Fischer UHPLC system (Thermo Fisher Scientific, California, USA) constituted of an ACCELA 1250 quaternary pump equipped with a degasser, a ACCELA autosampler equipped with column oven, and a Rheodyne valve with 25 *μ*L sample loop. Chromatographic separation was obtained using a Thermo Scientific Hypersil Gold PFP reversed-phase UHPLC column (100 mm, 2.1 mm ID, and 1.9 *μ*m). The LC eluents were water (A) and acetonitrile (B), everyone containing 0.1% (v/v) formic acid. The gradient was initiated with 70% eluent A and 30% eluent B for 0.5 min, continued with linear variation to 20% A and 80% B in 6.5 min; this condition was maintained for 0.5 min. The system returned to 70% A and 30% B in 0.5 min and was reequilibrated for 2 min. The column temperature was 30°C and the sample temperature was kept at 6°C. The flow rate was 0.4 mL min^−1^ and the injection volume was 5 *μ*L.

### 2.4. MS Conditions

The mass spectrometer was a triple quadrupole TSQ Vantage (Thermo Fisher Scientific, California, USA) in positive and negative electrospray ionization mode (ESI). Product ion scans of each analyte were performed by direct infusion (10 *μ*L min^−1^) of 1 mg L^−1^ individual standard solutions with the built-in syringe pump through a T-junction, mixing with the blank column eluate (200 *μ*L min^−1^).

ESI parameters optimized were as follows: capillary voltage 4.5 kV; capillary temperature 310°C; vaporizer temperature 150°C; sheath and auxiliary gas pressure were fixed at 35 and 10 (arbitrary units), respectively. The collision gas was argon at 1.5 mTorr and peak resolution of 0.7 FWHM was used on Q1 and Q3. The scan time for each monitored transition was 0.01 s and the scan width was 0.01 *m*/*z*. The collision energy parameters associated with the precursor and the product ions are given in Table 1. Acquisition data were recorded and elaborated using Xcalibur™ version 2.1.0.1139 software from Thermo.

### 2.5. Chromatography and Quantitative Determination

The presence of corticosteroids was verified by comparison between the chromatograms of the standard solution and the sample: the retention time and the relative abundances of the fragment were compared. For the quantitative determination, we used two methods: interpolation of the signal from the analytes of the sample in the solution calibration curve and the standard addition method. The calibration curve for the standard solutions was made with the concentration levels of 5-25-50-100-150-250 *μ*g L^−1^. For the standard addition method, we chose a cream containing a known amount of clobetasol propionate declared by the pharmaceutical company (0.05%, w/w). We made three spiked levels of concentration in order to have twice, three times, and five times the declared content of drug in the cream sample. The samples, thus obtained, were diluted, extracted, and analyzed. Then we calculated the concentration of the analyte by the extrapolation of the value through the intersection of the calibration curve and the *x*-axis. Areas used for the quantification are generated by the base peak signal only.

### 2.6. Validation Procedure

For the estimation of the validation parameters, blank samples were fortified at three different concentrations in equidistant steps: 0.5, 1.0, and 1.5 mg kg^−1^. Six spiked samples, at each of the three levels, were analyzed. The 18-replicate analyses (six for each level) were repeated in three separate days giving 54 independent determinations.

Linearity, specificity, recovery, matrix effect, limit of detection (LOD), limit of quantification (LOQ), precision (repeatability and the within-laboratory reproducibility), and accuracy were measured.

To test the selectivity/specificity of the method, 20 blank samples of different type (creams, gels, and ointments taken from make-up shops) were analyzed to verify the absence of potential interfering compounds at analytes retention time. Linearity was studied in the range of 6-point calibration curve for all the analytes. The recoveries were obtained using six replicates at each level. For the evaluation of matrix effects, three preparations were compared: the first is a blank sample spiked at 1.0 mg kg^−1^ and analyzed after the extraction procedure. The second is a blank matrix extract spiked immediately before LC injection. The third is a mix of the target analytes corresponding to spiked level.

Precision is expressed as the percent relative standard deviation (RSD%) of concentrations calculated for spiked samples and accuracy as the relative error of the calculated concentrations. Both parameters were measured in intra- and interday manner. The accuracy was tested also analyzing a pharmaceutical preparation containing a declared concentration of clobetasol propionate and comparing the results to the true concentration.

The limit of detection (LOD) was estimated on the basis of the results for six replicates of cream sample spiked at the 0.1 mg kg^−1^ level and was calculated using the formula: $LOD=\overset{-}{X}+3SD$, where $\overset{-}{X}$ is the mean of the calculated concentration and SD is the standard deviation of replicate analyses. The quantification limit (LOQ) was calculated using the formula: $LOQ=\overset{-}{X}+10SD$.

The calculated LOD and LOQ values are reported in Table 2.

## 3. Results and Discussion

### 3.1. Chromatography and Validation Results

The tunes of the MS conditions for standards and the deuterated ISs were performed by direct infusion of 1 mg L^−1^ individual standard solutions with the built-in syringe pump. It was found that the precursor ions with the most abundant signal are composed of the formate adduct, [M+HCOO]^−^, in electrospray negative mode; only for CLP was the most abundant signal obtained in positive mode monitoring the adduct [M+H]^+^. After that, we optimized the chromatographic conditions by several injections of a mixed solution of the target analytes at the concentration of 100 *μ*g L^−1^ in order to test different combinations of mobile phases. Then we found the best gradient condition, reported in the experimental section of this paper, for the best symmetry and resolution of the peaks. The spectrometric determination was performed in MRM mode in order to obtain better selectivity and sensitivity.

Method validation was performed by evaluating the following parameters: linearity, limit of detection (LOD), limit of quantification (LOQ), intraday variability (repeatability), interday variability (intermediate precision), and recovery (trueness). We chose as complex matrices different cosmetic products free of analytes, such as creams and ointments taken from make-up shops.

The presence of the target substances in cosmetic samples was validated by comparing the retention time of the peak areas to a high purity standard. Also the relative abundances of the mass transitions were used as identification parameter (Figures 2–11).

**Figure 12:**
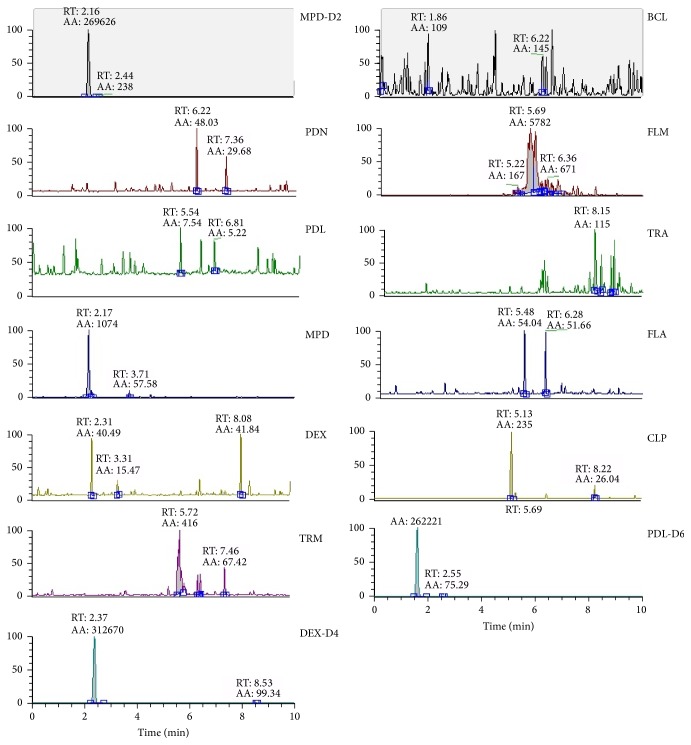
Chromatograms of the corticosteroids (PDN, PDL, MPD, TRM, BCL, FLM, TRA, FLA, DEX, and CLP) and internal standards (MPD-D2, DEX-D4, and PDL-D6) in the blank sample. The relative abundance (*y*-axis) is reported in percentage (%). AA: peak area; RT: retention time.

Specificity was demonstrated by identifying the analytes based on the precursor and product ions as well as the relative retention times (compared to the standards). Ion ratios in matrix-matched calibrators and analytes solutions typically matched each other to around 90%; hence, a maximum difference of 10% is tolerable. No interfering peaks at the RT of the analytes were found during selectivity test, consisting in a comparison between chromatogram of samples with standards in matrix and chromatogram of blank samples (Figure 12).

Linearity was studied within a concentration range of 5–250 *μ*g L^−1^ for all the steroids. The linearity ranges were fixed to secure the lower range limit [17].

Analytical method guarantees safety and efficiency because it highlights any corticosteroid residue. Any samples with levels above the linearity range can be appropriately diluted.

Calibration graphs were obtained including zero plotting the ratio analyte area/internal standard area (=*y*) versus analyte concentration (=*x*) for the analytes with the same deuterated standards and plotting the peak areas of analyte versus the corresponding concentration (*μ*g L^−1^ in the final dilution) for the others. A regression model was then applied to the calibration data set and linear calibration curves showed correlation coefficients *r*^2^ higher than 0.996. The high correlation coefficient *r*^2^ values indicated good correlations between corticosteroids concentrations and peak areas.

Standard addition method was also applied simultaneously to confirm the linearity and to determine drugs content. In the latter method, the concentration of steroids was determined by extrapolating the *x*-intercept value from the standard addition curve. This method was applied only to CLP, obtaining comparable results with the curve calibration method (within the range of 5%). The matrix effect was investigated in order to reveal possible ionization suppression or enhancement caused by matrix components. It was evaluated on different cosmetic products.

Two aliquots of each sample were extracted as previously described and the extracts were spiked of analytes and IS. At the same time, a solution of the detected analytes was prepared at the same concentration level. No significant ionic enhancement was found for each analyte and the absolute analytical recoveries obtained for spiked samples of three different concentrations were around 90%, excluding signal suppression or interferences due to endogenous substances in the complex matrix. LOD and LOQ values were found to be suitable for the purposes of the present study (Table 2). Particularly, the calculated LOQ tested for precision and accuracy presented RSD always lower than 20%. Excellent results were obtained for precision and accuracy of intraday and interday analyses with relative standard deviation (RSD) values within 10%, responding to established acceptance criteria [18, 19]. The internal standards were used as surrogate to measure the overall efficiency of the method (recovery) during its routine use. Trueness was expressed in terms of recovery rates; the values were in the range of 91–99%. Validation data are listed in Tables 2, 3, and 4.

### 3.2. Real Samples Analysis

The validated method has been applied to analyze 67 cosmetics samples, including 18 gels, 25 ointments, and 24 creams taken from the oily skin and seborrhea treatment courts of make-up shops in our territory. All samples were processed according to the method described. The samples were analyzed and found as not containing any of the monitored steroids.

## 4. Conclusions

In this work a LC-MS/MS method was validated. The method is accurate, precise, and suitable for the determination of ten different active substances of the glucocorticoids family in counterfeit cosmetic products. The extractive process has been proven to be rapid, efficient, and suitable for preparations such as creams and ointments. The chromatographic method allowed an optimal separation of the analytes; furthermore the MS/MS detection ensured a univocal identification and an excellent sensitivity. The proposed method that mainly aimed at the accurate and reproducible determination of ten steroids was found to be useful for the quality control of pharmaceutical formulations and the screening of counterfeit cosmetic products suspected to contain steroids, which are banned in cosmetics.

## Figures and Tables

**Figure 2:**
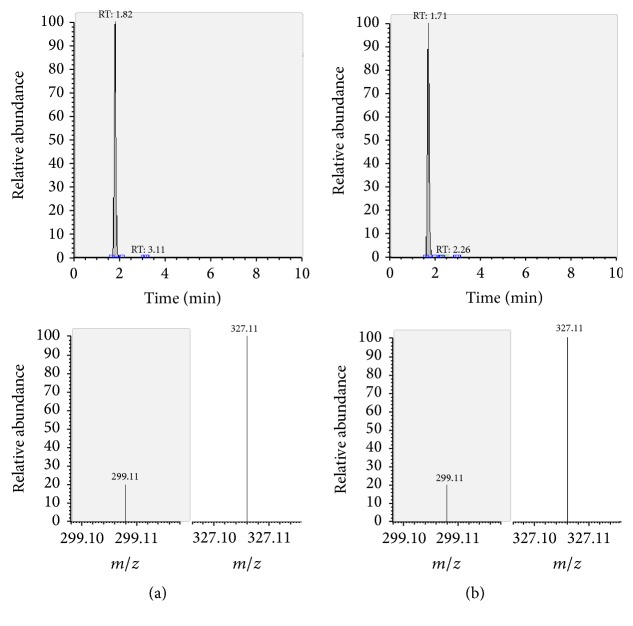
LC-MS/MS chromatograms of PDN and SRM transitions and relative abundances. (a) Standard in a 100 *μ*g L^−1^ solution. (b) Standard in the spiked sample at 100 mg kg^−1^.

**Figure 3:**
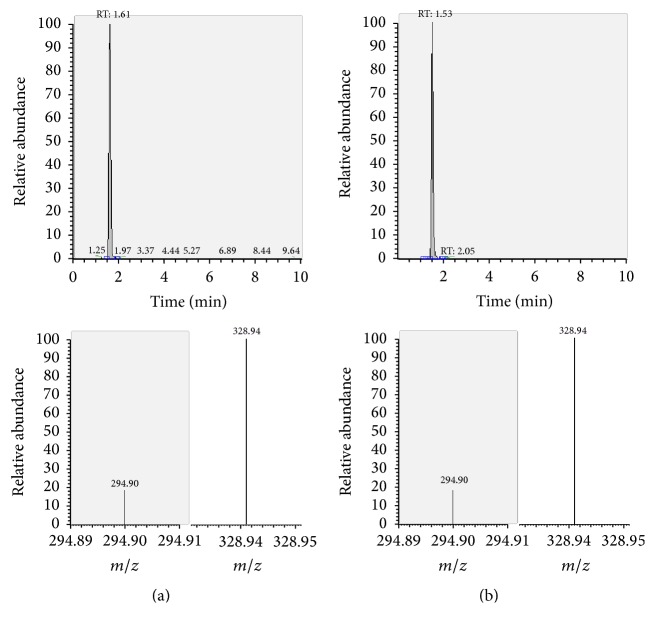
LC-MS/MS chromatograms of PDL and SRM transitions and relative abundances. (a) Standard in a 100 *μ*g L^−1^ solution. (b) Standard in the spiked sample at 100 mg kg^−1^.

**Figure 4:**
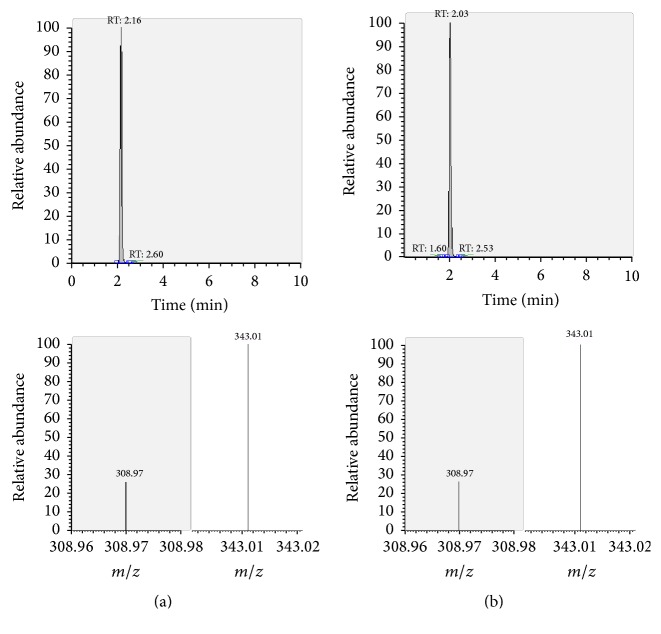
LC-MS/MS chromatograms of MPD and SRM transitions and relative abundances. (a) Standard in a 100 *μ*g L^−1^ solution. (b) Standard in the spiked sample at 100 mg kg^−1^.

**Figure 5:**
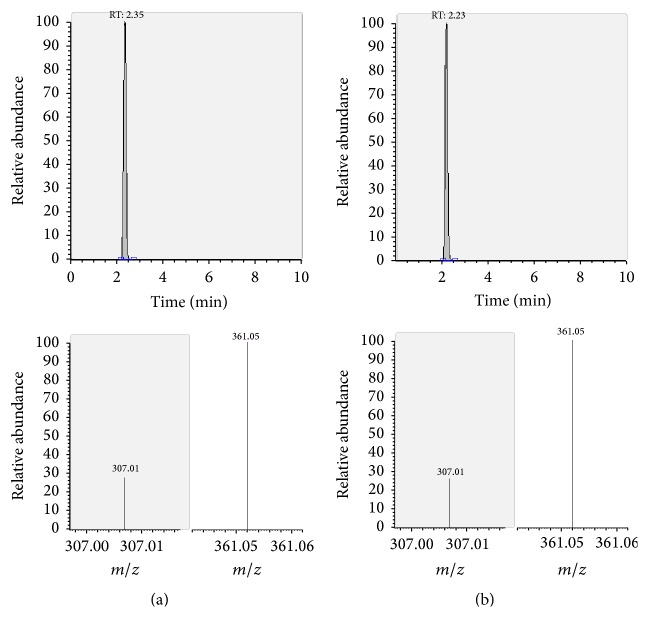
LC-MS/MS chromatograms of DEX and SRM transitions and relative abundances. (a) Standard in a 100 *μ*g L^−1^ solution. (b) Standard in the spiked sample at 100 mg kg^−1^.

**Figure 6:**
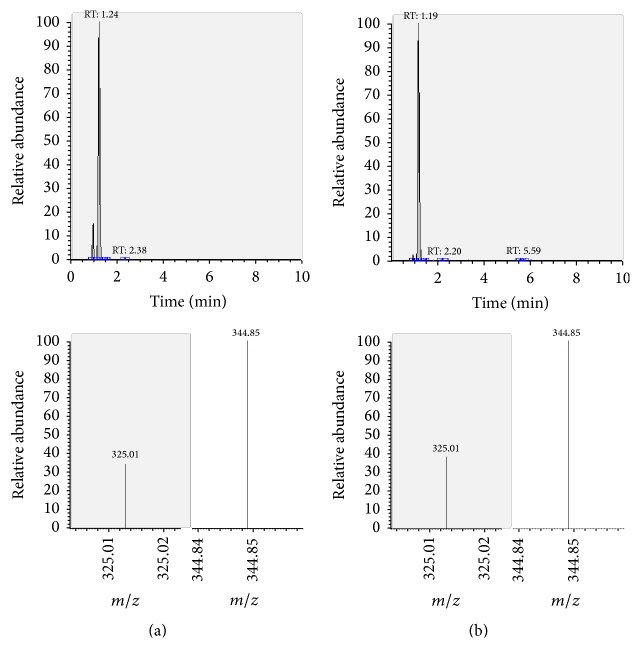
LC-MS/MS chromatograms of TRM and SRM transitions and relative abundances. (a) Standard in a 100 *μ*g L^−1^ solution. (b) Standard in the spiked sample at 100 mg kg^−1^.

**Figure 7:**
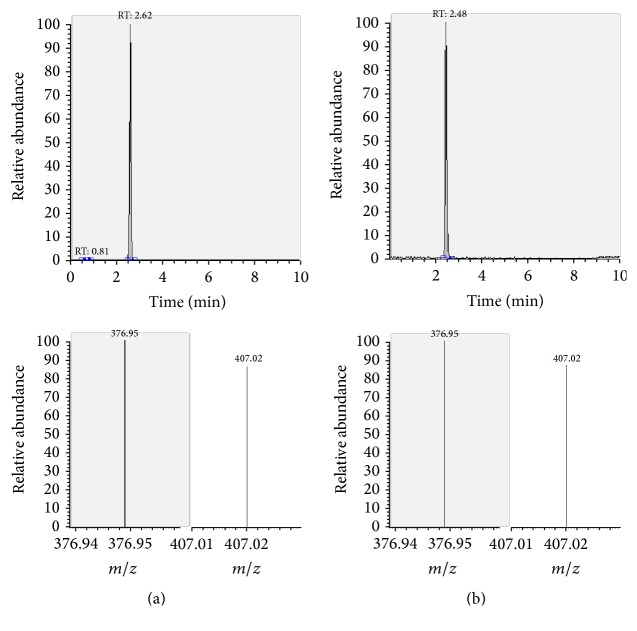
LC-MS/MS chromatograms of BCL and SRM transitions and relative abundances. (a) Standard in a 100 *μ*g L^−1^ solution. (b) Standard in the spiked sample at 100 mg kg^−1^.

**Figure 8:**
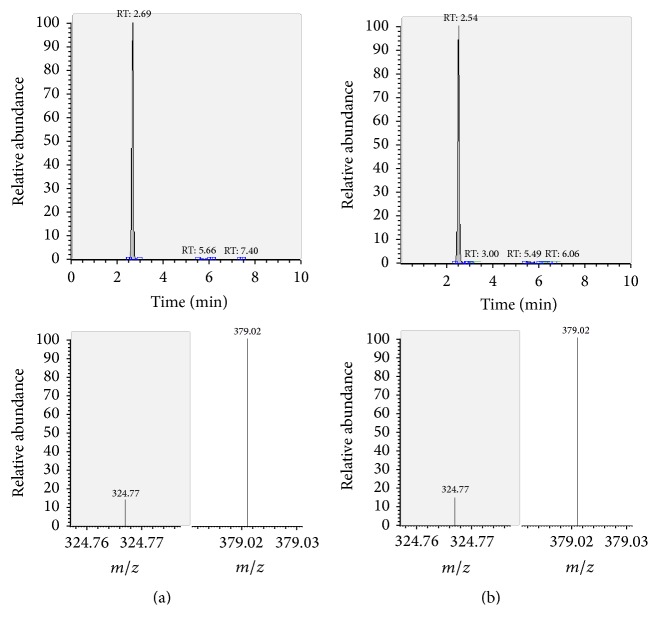
LC-MS/MS chromatograms of FLM and SRM transitions and relative abundances. (a) Standard in a 100 *μ*g L^−1^ solution. (b) Standard in the spiked sample at 100 mg kg^−1^.

**Figure 9:**
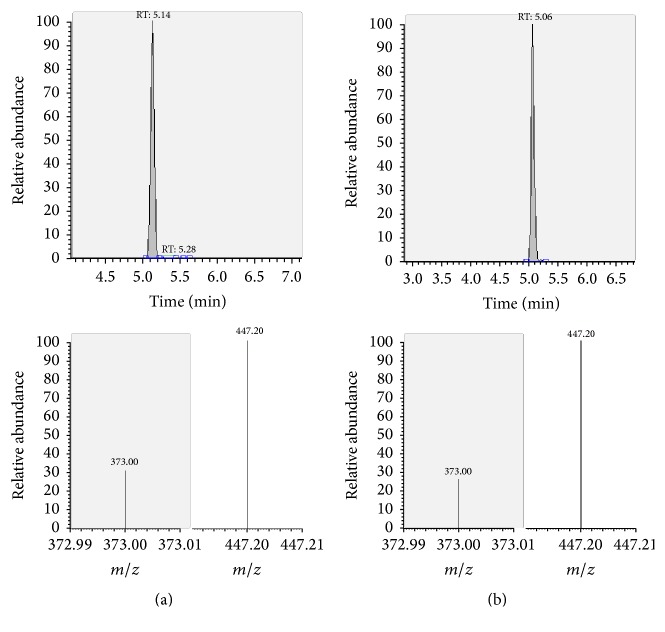
LC-MS/MS chromatograms of CLP and SRM transitions and relative abundances. (a) Standard in a 100 *μ*g L^−1^ solution. (b) Standard in the spiked sample at 100 mg kg^−1^.

**Figure 10:**
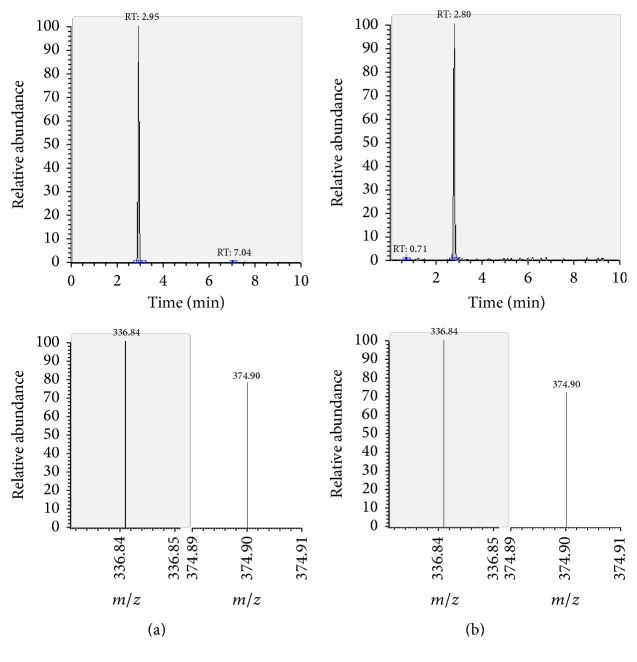
LC-MS/MS chromatograms of TRA and SRM transitions and relative abundances. (a) Standard in a 100 *μ*g L^−1^ solution. (b) Standard in the spiked sample at 100 mg kg^−1^.

**Figure 11:**
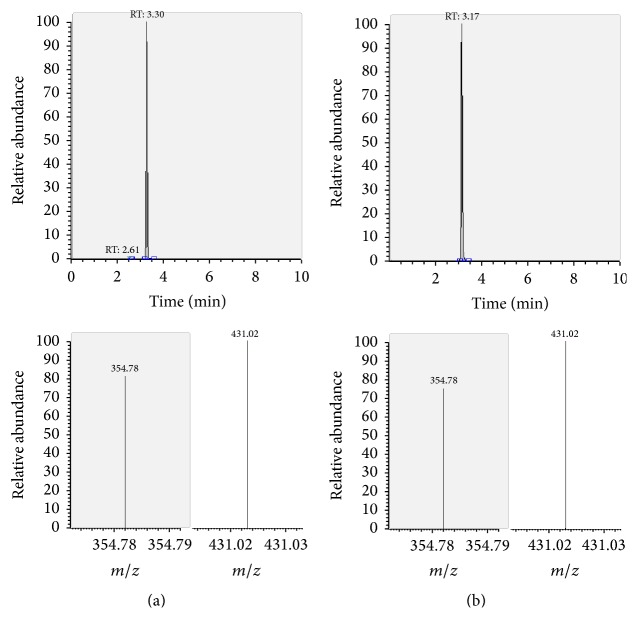
LC-MS/MS chromatograms of FLA and SRM transitions and relative abundances. (a) Standard in a 100 *μ*g L^−1^ solution. (b) Standard in the spiked sample at 100 mg kg^−1^.

**Table 1:** Parameters for SRM acquisition of the corticosteroids. ^a^The most abundant product ion.

Analyte	Internal standard	Retention time (min)	ESI	Precursor ion (*m*/*z*) [M+HCOO]^−^ or [M+H]^+^	Product ions (*m*/*z*)	Collision energy (eV)
MPD	MPD D2	2.16	neg	419.1	343.0^a^	20
			neg		308.9	32
DEX	DEX D4	2.35	neg	437.1	361.0^a^	19
			neg		307.0	30
PDL	PDL D6	1.61	neg	405.1	328.9^a^	19
			neg		294.9	32
FLA	—	3.30	neg	497.1	431.0^a^	21
			neg		354.8	22
FLM	—	2.69	neg	455.1	379.0^a^	19
			neg		324.8	30
PDN	—	1.82	neg	403.1	327.1^a^	17
			neg		299.1	20
TRM	—	1.24	neg	439.1	344.9^a^	23
			neg		325.0	24
TRA	—	2.95	neg	479.1	374.9^a^	25
			neg		336.8	19
BCL	—	2.62	neg	453.0	407.0^a^	14
			neg		376.9	16
CLP	—	5.14	pos	467.2	447.2^a^	10
			pos		373.0	12
DEX D4		2.38	neg	441.1	363.2	20
MPD D2		2.17	neg	421,1	342.8	19
PDL D6		1.61	neg	411.1	333.0	18

**Table 2:** Validation data of linearity and LOD and LOQ for samples containing spiked standard solutions in blank cosmetic preparations.  ^a^Internal calibration.  ^b^Concentration in mg kg^−1^.

Steroids	Standard solutions			
	Slope	*r* ^2^	LOD^b^	LOQ^b^
CLP^a^	0.008	0.999	0.089	0.102
DEX^a^	0.058	0.999	0.109	0.117
MPD^a^	0.062	0.998	0.103	0.121
PDL^a^	0.055	0.999	0.085	0.099
FLA	1553	0.996	0.093	0.107
FLM	13892	0.998	0.097	0.105
PDN	4123	0.999	0.098	0.112
TRM	1741	0.998	0.092	0.101
TRA	1635	0.997	0.101	0.113
BCL	1986	0.998	0.104	0.117

**Table 3:** Validation data of recovery for samples containing spiked standard solutions in blank cosmetic preparations.

Steroids	Spiked conc. (mg kg^−1^)	% recovery
CLP	0.5	98.9
	1.0	99.0
	1.5	99.1

DEX	0.5	94.3
	1.0	96.2
	1.5	96.8

MPD	0.5	98.8
	1.0	97.6
	1.5	99.1

PDL	0.5	95.5
	1.0	96.3
	1.5	97.7

FLA	0.5	92.1
	1.0	93.4
	1.5	92.9

FLM	0.5	97.6
	1.0	99.1
	1.5	98.8

PDN	0.5	99.2
	1.0	99.5
	1.5	99.0

TRM	0.5	93.1
	1.0	92.6
	1.5	94.2

TRA	0.5	92.4
	1.0	91.9
	1.5	93.0

BCL	0.5	96.4
	1.0	93.4
	1.5	94.7

**Table 4:** Validation data of precision and accuracy for samples containing spiked standard solutions in blank cosmetic preparations.  ^a^RSD (%) = (SD/mean *C*_obs_) × 100.  ^b^Accuracy (Bias%) = [(*C*_obs_ − *C*_nom_)/*C*_nom_] × 100.

Steroids	Intraday analysis (*n* = 6)						Interday analysis (*n* = 18)					
	Precision^a^			Accuracy^b^			Precision^a^			Accuracy^b^		
	0.5	1.0	1.5	0.5	1.0	1.5	0.5	1.0	1.5	0.5	1.0	1.5
CLP	2.8	4.2	3.9	1.0	1.2	0.9	3.4	5.1	4.8	0.9	1.0	1.1
DEX	3.1	4.1	6.1	2.8	2.4	2.1	4.2	5.3	6.9	1.9	1.8	2.4
MPD	3.5	2.3	2.3	4.4	3.8	3.7	3.9	2.9	3.1	4.2	3.8	3.6
PDL	2.6	3.4	2.4	1.2	1.4	1.2	2.1	3.9	2.9	1.8	1.5	1.1
FLA	5.1	4.0	5.7	2.0	1.7	1.5	4.7	4.5	6.1	1.9	1.7	1.1
FLM	4.0	3.5	2.6	2.7	1.8	1.9	5.2	4.7	4.5	1.9	1.8	1.9
PDN	4.7	4.3	3.3	2.6	2.3	2.2	5.1	4.7	3.1	3.1	2.8	2.2
TRM	2.9	3.9	2.6	3.4	2.8	2.2	3.3	4.2	3.8	3.8	3.8	4.2
TRA	5.2	4.9	4.8	0.8	0.9	1.0	5.9	6.1	6.2	1.2	1.4	1.1
BCL	6.1	5.9	5.6	6.0	5.4	5.2	6.5	6.3	6.8	4.5	6.1	5.0
